# The effect of anastrozole therapy on final height and sex hormone levels in pubertal boys receiving growth hormone therapy

**DOI:** 10.20945/2359-4292-2022-0524

**Published:** 2023-12-01

**Authors:** Gürkan Tarçın, Cansu Koç, Hande Turan, Oya Ercan

**Affiliations:** 1 Istanbul University-Cerrahpaşa Cerrahpaşa Faculty of Medicine Department of Pediatric Endocrinology Istanbul Türkiye Istanbul University-Cerrahpaşa, Cerrahpaşa Faculty of Medicine, Department of Pediatric Endocrinology, Istanbul, Türkiye

**Keywords:** Anastrozole, final height, growth hormone, pubertal boy

## Abstract

**Objective:**

This research aimed to evaluate retrospectively the effect of anastrozole on height gain and sex hormone levels in pubertal boys receiving growth hormone (GH).

**Materials and methods:**

Pubertal boys who received both GH and anastrozole (GH+A) were one-to-one matched with boys who received only GH (GH-Only) for chronological and bone age, pubertal stage and height before the GH initiation, treatment duration and midparental height. Anthropometric measurements throughout treatment and adult heights were compared between the groups. Sex hormone levels were evaluated longitudinally in the GH+A group.

**Results:**

Forty-eight cases (24 in each group) were included. There was no statistical difference in adult height between the GH+A and GH-Only (p = 0.071). However, when the analysis was limited to those receiving anastrozole for at least 2 years, mean adult height was higher in the GH+A than in the GH-Only group (173.1 ± 6.2/169.8 ± 5.6 cm, p = 0.044). Despite similar growth rates between the two groups, bone age advancement was slower in the GH+A than in the GH-Only in a mean anastrozole treatment period of 1.59 years (1.37 ± 0.80/1.81 ± 0.98 years, p = 0.001). The greatest increase for FSH, LH, total and free testosterone and decrease for estradiol levels were observed in the third month after anastrozole was started, albeit remaining within the normal ranges according to the actual pubertal stages.

**Conclusions:**

Using anastrozole with GH for at least 2 years decelerates the bone age advancement resulting in adult height gain with no abnormality in sex hormone levels. These results suggest anastrozole can be used as an additional treatment to GH for further height gain in pubertal boys.

## INTRODUCTION

In children receiving growth hormone (GH) therapy during puberty, epiphyseal fusion due to the effect of sex steroids often limits the height gain ( 1 ). The senescence of the growth plate is irreversible and depleted progenitor cells in resting chondrocytes causes it. Estrogen is the main hormone that accelerates this process in both genders ( 2 ). Although using gonadotropin-releasing hormone (GnRH) analogues can delay epiphyseal fusion by decreasing estrogen levels, it causes hypogonadism during a critical period of sexual maturation. It also has some metabolic effects, such as a decrease in protein synthesis and an increase in adiposity and urinary calcium excretion ( 1 , 3 , 4 ), and rarely, pseudotumor cerebri has been reported as a significant adverse event ( 5 ). At this point, aromatase inhibitors (AIs), which block the conversion of androgens to estrogens, may seem a better alternative. However, clinical use of AIs in this regard is not common.

Studies examining the effect of AIs on height gain have come to the fore especially in the last decade, although they are still few in number ( 6 – 10 ). Conflicting results have been reported in studies regarding the effect of AIs (letrozole or anastrozole) on height gain in pubertal males. While some studies indicated that using AIs did not increase height gain or adult height ( 6 – 8 ), other studies have shown benefits ( 9 , 10 ). Thus, the efficacy of AIs alone or combined with GH on height gain needs to be investigated further.

This study aimed to investigate the efficacy of anastrozole for additional treatment in pubertal male patients receiving GH therapy as well as the alterations in sex hormone levels during the treatment.

## MATERIALS AND METHODS

### Study design and subject selection

This is a retrospective study comparing two groups of male patients treated with GH either with or without anastrozole. The medical records of all male patients who had received GH and achieved adult height between 2006 and 2021 were reviewed. Patients with systemic disorders (e.g., celiac disease, hypothyroidism), skeletal dysplasia, syndromic growth disorders or low birth weight (SGA) were excluded. All patients had been followed up for at least 6 months before GH treatment began, and their growth velocities were found to be low (below the 25^th^ percentile). Afterwards, two growth hormone stimulation tests were performed using L-DOPA and clonidine. The cut-off value for GH deficiency was considered as 10 ng/mL. GH therapy was started in all patients who were either GH deficient or proved not to have GH insensitivity by an insulin-like growth factor (IGF) generation test ( 11 ). Anastrozole treatment was offered to the pubertal patients whose bone age was found to be advancing rapidly (at a faster rate than the actual passage of time) while receiving GH with a low predicted adult height for the midparental height (midparental height: 7 cm).

### Follow-up data

Quarterly anthropometric measurements, pubertal examinations, annual bone age and GH stimulation tests were obtained from the patients’ medical files. The same clinician determined bone age according to Greulich and Pyle's ( 12 ) method. In patients receiving anastrozole, follicle stimulating hormone (FSH), luteinizing hormone (LH), estradiol and total and free testosterone were measured every 3 months during the treatment. These levels were interpreted according to the normal ranges specified for the pubertal stage ( 13 , 14 ). GH was given at a dose of 18 IU/m^2^/week and anastrozole at a dose of 1 mg/day. The patients’ adult heights were obtained from the medical records, if not available, by calling them and taking their current measurements.

### Anthropometric measurements and physical examination

Height was measured with a stadiometer (Holtain Limited, Crymych, United Kingdom) with the subject naked feet and back against a backboard. Standard deviation score (SDS) of height was calculated according to the standards for Turkish Children ( 15 , 16 ). Predicted adult height was calculated using the Bayley-Pinneau tables ( 17 ). The pubertal status of all subjects was assessed according to the Tanner staging ( 18 ).

### Creating the study groups

To evaluate the effect of anastrozole on height gain in patients receiving GH therapy, two groups were formed: 1) The subjects who received anastrozole treatment for at least 1 year during GH treatment (GH+A) and 2) the subjects who received only GH treatment (GH-Only) selected from those with closely similar midparental height, baseline chronological and bone age, pubertal stage, height and height SDS to those in the GH+A group to create a matched group.

**Figure 1:**
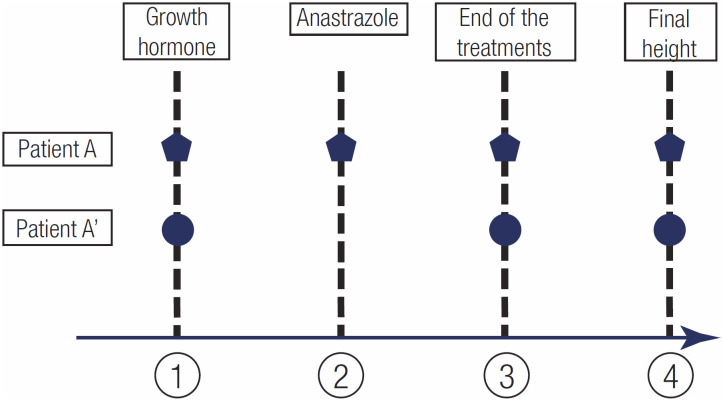
Study design. Patient A and A’ represent matched pairs who have the same baseline characteristics receiving GH+A and GH-Only therapy, respectively. The numbers represent the milestones as follows: 1. initiation of GH therapy, 2. addition of anastrozole for patient A (for patient A’, measurements were noted at the same chronological age as patient A [TIME 2’]), 3. cessation of the treatments, and 4. final height.

Chronological age, bone age, pubertal stage, height and height SDS were noted from the medical records of the patients who received GH+A therapy at 3 different times: at the beginning of GH (TIME 1), when anastrozole was added (TIME 2) and at cessation of both GH and anastrozole (TIME 3). Adult height was also noted in all patients (TIME 4). Because there was no such milestone as TIME 2 in the GH-Only group, for each patient, the findings were noted at the corresponding TIME 2 (TIME 2’: the same chronological age of each patient's partner in the GH+A group when anastrozole was added). The study design is schematized in Figure 1 .

### Statement of ethics

The local ethical committee approved the study protocol (date and number: 25.06.2021/004).

### Statistical analyses

Statistical analyses were performed using the Statistical Package for Social Sciences (SPSS) software for Windows, version 22 (Chicago, Illinois). The variables were investigated using visual (histograms) and analytical methods (Shapiro-Wilk's test) to determine if the data were normally distributed. For paired data, paired sample t-test or Wilcoxon test was used, and Student's t-test or Mann-Whitney U-test was used in the independent groups based on their distribution status. A p value of < 0.05 was considered statistically significant.

## RESULTS

**Figure 2:**
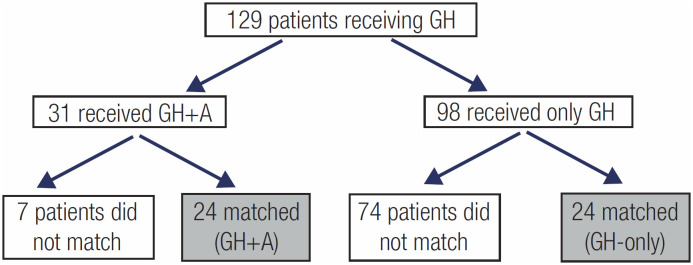
Formation of the GH-Only and GH+A groups.

**Figure 3:**
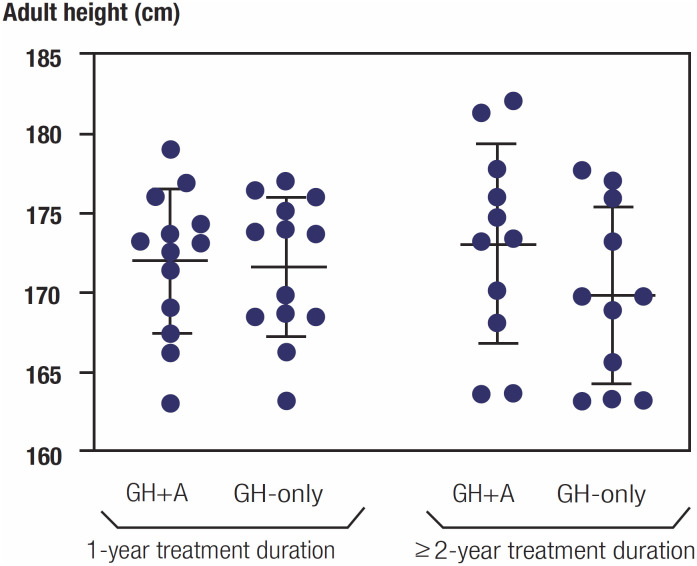
The adult heights of the patients grouped by anastrozole treatment duration in the study (GHA1 and GHA2).

There were 129 male patients who had received GH therapy and reached their adult height. Thirty-one of these agreed to use anastrozole and received it for at least 1 year during GH therapy, while the remaining 98 patients were not offered or did not agree to use it. When the 31 patients who used GH+A were matched in terms of the paired parameters (chronological age, bone age, pubertal status and height at the start of GH, duration of GH treatment and midparental height) with the 98 patients who used only GH, 7 of them did not have a pair in the GH-Only group resulting in excluding them from the study. Thus, a total of 48 cases (24 cases in each group) were included in the study ( Figure 2 ). There was no statistical difference between the groups regarding the paired parameters before the start of GH ( Table 1 ). In the GH+A group, anastrozole was added an average of 1.5 years after the start of GH treatment. At the milestone when anastrozole was started (TIME 2), the similarity was maintained between the GH-only and GH+A groups in terms of age, height, height SDS and bone age (14.0 ± 0.9/14.0 ± 1.0 years, p = 0.489; 154.04 ± 6.32/154.19 ± 6.38 cm, p = 0.820; −1.34 ± 0.37/−1.29 ± 0.45 SDS, p = 0.588; 13.0 ± 1.0/13.1 ± 0.7 years, p = 0.455, respectively). Thirteen cases received anastrozole treatment for 1 year, 8 for 2 years, 2 for 2.5 years, and 1 for 4 years. Anastrozole and GH treatment were discontinued simultaneously in all cases. There was no significant difference between the GH-Only and GH+A groups regarding adult height (p = 0.071). However, when the entire group was divided into two groups according to the treatment duration (those who received anastrozole treatment for 1 year [GHA1] and at least 2 years [GHA2]), there was no difference in adult height in the GHA1 group (n = 13) compared to their pairs in the GH-Only group (p = 0.730); whereas in the GHA2 group (n = 11), the adult height was significantly higher with an average of 3.3 cm (173.1 ± 6.2 *vs.* 169.8 ± 5.6 cm, p = 0.044) ( Table 2 ). In Figure 3 , a graphic shows the adult height of each individual in the GHA1 and GHA2 groups.

**Table 1:** The characteristics of the patients who received GH+A and GH-Only at the onset of GH treatment

		GH+A (n = 24)	GH-Only (n = 24)	p
CA (years)		12.4 ± 1.2	12.5 ± 1.1	0.318
Height (cm)		141.7 ± 7.9	141.5 ± 7.7	0.727
Height SDS		−1.6 ± 0.5	−1.7 ± 0.5	0.428
BA (years)		11.4 ± 1.4	11.2 ± 1.4	0.479
Predicted adult height (cm)		170.3 ± 3.7	170.4 ± 4.5	0.953
Midparental height (cm)		171.1 ± 4.2	170.4 ± 3.9	0.174
Pubertal status				
	Tanner 2	n = 15	n = 15	
	Tanner 3	n = 6	n = 6	
	Tanner 4	n = 3	n = 3	
Duration of GH treatment (year)		3.2 ± 1.3	3.1 ± 1.2	0.339

CA: chronological age, SDS: standard deviation score, BA: bone age, GH: growth hormone, A: anastrozole.

**Table 2:** Comparison of the baseline characteristics and treatment outcomes in patients who received GH+A and GH-Only according to treatment duration

	The patients who received anastrozole for 1 year (n = 13)			The patients who received anastrozole for ≥ 2 years (n = 11)		
	GH+A	GH-Only	P	GH+A	GH-Only	p
Age at the start of GH treatment (TIME1) (year)	12.5 ± 1.2	12.6 ± 1.1	0.588	12.1 ± 1.1	12.2 ± 1.2	0.318
Height at the start of GH treatment (TIME1) (cm)	141.9 ± 7.9	141.8 ± 8.0	0.826	141.3 ± 8.3	141.1 ± 7.7	0.721
Height SDS at the start of GH treatment (TIME1)	−1.75 ± 0.59	−1.79 ± 0.55	0.727	−1.48 ± 0.50	−1.58 ± 0.41	0.438
Total duration of GH treatment (year)	2.8 ± 1.5	2.8 ± 1.3	0.929	3.7 ± 0.9	3.5 ± 1.0	0.072
ΔHeight (cm)	8.4 ± 2.8	7.8 ± 2.9	0.408	15.6 ± 4.0	14.24 ± 4.9	0.231
ΔHeight SDS	0.46 ± 0.31	0.34 ± 0.39	0.327	0.52 ± 0.61	0.33 ± 0.57	0.346
ΔBA (year)	1.02 ± 0.46	1.27 ± 0.46	0.059	1.79 ± 0.92	2.45 ± 1.05	0.003
Height at the end of the GH (and A) treatment (cm) (TIME3)	163.8 ± 5.7	164.5 ± 4.7	0.501	168.3 ± 6.2	165.1 ± 4.5	0.073
Adult height (cm)	172.0 ± 4.5	171.6 ± 4.3	0.730	173.1 ± 6.2	169.8 ± 5.6	0.044
Adult height corrected by midparental height (cm)	1.25 ± 4.66	0.53 ± 4.51	0.581	1.64 ± 5.81	0.20 ± 5.58	0.247

SDS: standard deviation score, BA: bone age, GH: growth hormone, A: anastrozole Δ values represent the difference between the beginning and end of anastrozole treatment for the GH+A group and the corresponding times for the GH-Only group (TIME3-TIME2).

There was no significant difference in bone age and height between the GH-Only and GH+A groups at the beginning of anastrozole treatment (p = 0.489 and p = 0.820, respectively). Even though the growth rate during anastrozole therapy and the height reached at the end of the treatment (TIME 3) were similar between the GH+A and GH-Only groups (p = 0.133 for growth rate and p = 0.269 for the height at the end of the treatment), advancement in bone age was slower in the GH+A group than in the GH-only group during the same chronological period of anastrozole therapy (1.37 ± 0.80/1.81 ± 0.98 years in a mean period of 1.59 years, p = 0.001). No side effects were observed in any of the patients during anastrozole treatment.

**Figure 4:**
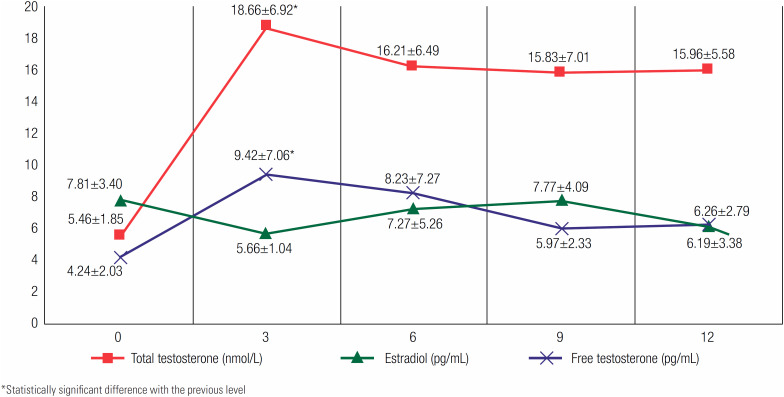
The mean levels of total testosterone, free testosterone and estradiol during anastrozole treatment at 3-month intervals.

**Figure 5:**
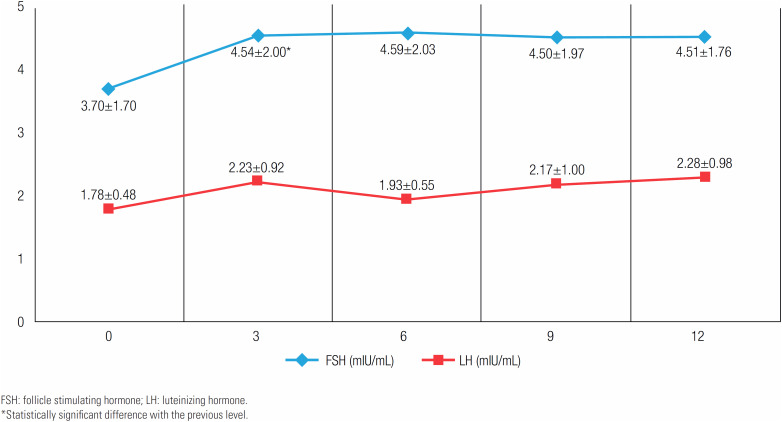
The mean levels of gonadotropins during anastrozole treatment at 3-month intervals.

Figures 4 and 5 show the mean levels of each hormone over a 1 year period at 3-month intervals following the addition of anastrozole therapy. The greatest change in all measured hormones was observed in the first 3 months. The difference between baseline and at 3 months was significant in FSH, total testosterone and free testosterone levels (p = 0.048, p < 0.001 and p = 0.042, respectively). During treatment, these levels nevertheless remained within the normal ranges appropriate for pubertal stage.

## DISCUSSION

This study's main finding was that at least 2 years of anastrozole use with GH resulted in a significant increase in adult height in pubertal males. However, there was no significant difference between the two similar midparental heighted and bone aged groups regarding their height both at the beginning and at the end of the treatments. The progression in bone age was slower in the group that received anastrozole, which likely explains the gain in the adult height. In addition, during anastrozole treatment, sex hormone levels did not reach abnormal levels.

The limited number of studies on the efficacy of AIs on height gain have conflicting results, as previously stated ( 6 – 10 ). Furthermore, in numerous comparative studies, a concomitant treatment with GH showed to increase adult height ( 1 , 19 , 20 ). In Mauras and cols.'s ( 19 ) well-designed prospective study, 52 pubertal boys with GH deficiency treated with GH were randomized to cotreatment with anastrozole or placebo for up to 36 months. While the net gains in predicted adult height were: +1.3, +4.5 and +6.7 cm in those treated with anastrozole for 12, 24 and 36 months, respectively, these values in the group that received placebo were +0.3, +1.1 and +1.0 cm, respectively. The researchers concluded that estrogen blockade provided an advantage after a period of at least 2 years, and the difference in the predicted adult height between the two groups at the second year was 3.4 cm, which is very close to our study's finding (a difference of 3.3 cm) despite being based on only 11 patients. However, while our results are based on measured adult heights, Mauras and cols.'s ( 19 ) reported values were based on predicted adult height. The same researcher ( 1 ) conducted another study with 76 pubertal males – this time with idiopathic short stature – randomized to 3 arms according to the treatment: AI (either letrozole or anastrozole), GH or a combination of GH and AI. At baseline, chronological age, bone age, height and midparental height were similar in all 3 groups, and at the end of 24 months of treatment, the greatest mean height gain was in the group that received GH and anastrozole together, then GH alone, then only AI (+18.9, +17.1 and +14.0 cm, respectively). These patients were followed up until near-adult height, and although the mean near-adult heights of the groups followed the same order, no statistically significant difference could be found among them. A French group ( 21 ) conducted a similar study with males with ISS in the late stages of puberty. Twenty-four boys (12 started on only GH and 12 had a combination of GH and anastrozole treatment) had a mean chronological age of 15.2 years, and mean bone age of 14.5 years. Despite the late initiation of the AI, the study demonstrated a significant difference between adult heights in favor of the group that received anastrozole and GH treatment (168.4 ± 2.6 cm *vs.* 164.2 ± 5.6 cm, and height increments during treatment were 12.7 ± 5.6 cm *vs.* 7.8 ± 5 cm). Lastly, a longitudinal cohort study concluded that adding an AI improved height SDS in pubertal boys with idiopathic short stature or GH deficiency receiving GH therapy ( 20 ). At the initiation of AI therapy, height SDSs were −0.92 ± 0.89 in the boys with GHD (n = 70) and −0.87 ± 0.86 in the boys with ISS (n = 19). After 1 year of therapy, these values increased to −0.62 ± 0.95 and −0.69 ± 0.83, respectively, while bone age to chronological age ratio decreased (which heralds a higher adult height) in both groups in the meantime. However, unlike our study, the subjects’ adult heights were not provided. Additionally, the data were longitudinal and not compared with a control group using only GH. Table 3 summarizes studies that are similar to ours as well as our study.

**Table 3:** Summary of the data from similar studies, including this study

Group	n	Baseline			AI treatment duration	Treatment outcome (cm) (AH)	Conclusion
		CA (year)	Height (cm) ± SDS	BA (year)			
**This study**							
GH	24	12.5 ± 1.1	141.5 ± 7.7 −1.7 ± 0.5	11.2 ± 1.4	1.59 ± 0.77 years	171.6 ± 4.3 a 169.8 ± 5.6 b	Combining AI with GH therapy for ≥ 2 years provides a significant gain in AH.
GH+AI	24	12.4 ± 1.2	141.7 ± 7.9 −1.6 ± 0.5	11.4 ± 1.4		172.0 ± 4.5 a 173.1 ± 6.2 b	
**Mauras, 2016 (** 1 **)**							
GH	25	14.1 ± 0.1	144.2 ± 1.4 (−2.4 ± 0.1)	12.9 ± 0.3	2 to 3 years	164.8 ± 1.6 (−1.4 ± 0.2)	GH+AI treatment for 2 years increases height potential, which can be further improved with 3 years of treatment.
GH+AI	26	14.0 ± 0.2	144.5 ± 1.3 (−2.3 ± 0.1)	12.7 ± 0.2		166.9 ± 1.5 (−1 ± 0.1)	
**Mauras, 2008 (** 19 **)**							
GH	26	14.2 ± 0.2	151.6 ± 1.3 (−1.5 ± 0.2)	13.4 ± 0.2	3 years	+0.3 ± 1.0 c +1.1 ± 1.1 d +1.0 ± 1.1 e	2-3 years of aromatase blockade in GH-treated boys results in a significant gain in predicted adult height.
GH+AI	26	13.8 ± 0.3	149.7 ± 1.6 (−1.4 ± 0.2)	13.7 ± 0.2		+1.3 ± 0.7 c +4.5 ± 1.2 d +6.7 ± 1.4 e	
**Rothenbuhler, 2015 (** 21 **)**							
GH	12	15.2 ± 1.1	156.3 ± 2.9 (−1.7 ± 1.0)	14.6 ± 0.6	19 ± 5.9 months	164.2 ± 5.6 (−1.8 ± 0.9)	Administration of GH and AI can increase AH in adolescents in their late stage of puberty.
GH+AI	12	15.2 ± 0.8	155.9 ± 4.0 (−1.7 ± 0.7)	14.5 ± 0.2		168.4 ± 2.6 (−1.1 ± 0.4)	

ᵃ and ^b^ represent the adult heights of the subgroups receiving anastrozole treatment for 1 and ≥ 2 years, respectively.

^c^, ^d^ and ^e^ represent the gains in predicted adult height in the 1^st^, 2^nd^ and 3^rd^ years, respectively. Data regarding adult heights were not provided in the study.

AH: adult height; AI: aromatase inhibitor; BA: bone age; CA: chronological age; GH: growth hormone; SDS: standard deviation score.

When the levels of gonadotropins and sex steroids after the addition of anastrozole treatment were examined, an increase in FSH, LH, total and free testosterone was found, whereas a decrease in estradiol levels inversely to the changes in the levels of total and free testosterone was observed. The greatest changes in these hormones’ levels were observed in the 3^rd^ month after initiating anastrozole, albeit remaining within the normal ranges appropriate for the pubertal stages of the patients throughout the treatment. Given that the aromatase enzyme converts androgens to estrogens, it is expected that testosterone levels will increase and estrogen levels will decrease in case of its inhibition. Estradiol has a strong effect on gonadotropin release in both males and females. A decrease in estradiol level due to an AI is associated with the release of FSH and LH at the pituitary level. This increase also contributes to the rise in testosterone level ( 22 , 23 ). These changes in gonadotropin and sex steroids have brought safety concerns. In a study conducted in patients with idiopathic short stature, serum testosterone levels reached supraphysiological levels as a result of increased LH levels during letrozole treatment ( 10 ). In another study with anastrozole ( 19 ), this increase in testosterone was less, which was probably due to its lower potency than letrozole. This increase may accelerate the development of physical signs of puberty ( 24 ). However, in the current study, testosterone level, despite an apparent increase, did not exceed the upper limit for the given pubertal stage in any patient.

Although this study's retrospective design may be considered a limitation, the one-to-one match between patients receiving GH+A and GH-Only in terms of the characteristics that would affect the study's outcome makes this study powerful and provides an opportunity to reflect on the effect of anastrozole. Another strength of this study is that while studies on this subject mostly provide data on predicted adult height, adult heights are provided in this study, which makes the results more reliable. Besides, as an additional advantage, the same clinician performed all patients’ physical examinations and bone age determinations. However, notably, the study's results are based on the treatment outcomes of children who have heights close to the lower limit of the reference range. Although these results provide valuable insights, further investigation is needed to determine the potential benefits of anastrozole treatment for children with short stature and poor predicted adult height.

In conclusion, in this study, the addition of anastrozole treatment for a minimum of 2 years in children receiving growth hormone resulted in a small yet non-trivial gain in adult height, while not affecting the growth rate but slowing down the advancement of bone age. Even though the findings of this retrospective study are based on a limited number of patients, a meticulous analysis of our data supports that cotreatment of GH and anastrozole could be used in pubertal male patients to achieve a better adult height without causing noteworthy changes in sex hormone levels. Nevertheless, it is important to note that the gain in adult height can vary for each patient, and parents should be informed about this variability when considering anastrozole therapy in clinical practice.
